# 超高效液相色谱-串联质谱法直接测定水中5种黄原酸

**DOI:** 10.3724/SP.J.1123.2022.09002

**Published:** 2023-04-08

**Authors:** Weihong ZHU, Chao WANG, Linlin ZHANG, Mao YUAN

**Affiliations:** 1.中国环境监测总站, 北京 100012; 1. China National Environmental Monitoring Centre, Beijing 100012, China; 2.宝鸡市环境监测中心站, 陕西 宝鸡 721000; 2. Baoji Environmental Monitoring Station, Baoji 721000, China

**Keywords:** 超高效液相色谱-串联质谱法, 直接进样, 丁基黄原酸, 同分异构体, 黄药, 地表水, 地下水, 工业废水, ultra performance liquid chromatography-tandem mass spectrometry (UPLC-MS/MS), direct injection, butyl xanthic acid, isomer, xanthate, surface water, ground water, industrial sewage

## Abstract

建立了超高效液相色谱-串联质谱法测定水中乙基黄原酸、异丙基黄原酸、正丁基黄原酸、异丁基黄原酸和戊基黄原酸等5种黄原酸的分析方法。水样经0.22 μm亲水聚四氟乙烯(PTFE)滤膜过滤后直接进样分析,采用Waters Acquity UPLC BEH C_18_色谱柱(100 mm×2.1 mm, 1.7 μm)进行分离,以氨水溶液(pH 11)-乙腈(9∶1, v/v)作为流动相进行等度洗脱,多反应监测负离子模式测定,内标法定量。通过将流动相氨水溶液的pH值增加到11,可有效抑制黄原酸色谱峰的拖尾现象,从而改善分离效果,并使丁基黄原酸同分异构体得到分离。水样保存条件确定为pH 11、4 ℃避光保存,在该条件下保存期限可延长至8 d。5种黄原酸在0.25~100 μg/L范围内线性关系良好,方法检出限为0.03~0.04 μg/L,日内精密度和日间精密度分别为1.3%~2.1%和3.3%~4.1%。低、中、高加标水平(1.00、20.0、80.0 μg/L)下的回收率分别为96.9%~133%、100%~107%和104%~112%,对应的相对标准偏差分别为2.1%~3.0%、0.4%~1.9%和0.4%~1.6%。优化后的方法可成功用于地表水、地下水和工业废水的分析。该方法无需繁琐的前处理过程,具有进样量少、操作简单、灵敏度高、水样保存时间久等优点,适用于水中多种黄原酸的同时分析。

## Abstract (en)

Xanthates with different alkyl groups, such as ethyl, propyl, butyl, and amyl groups, are widely used in large quantities in the mining flotation of metallic minerals. Xanthates enter environmental waters through mineral processing wastewater discharge and are ionized or hydrolyzed into ions or molecules of xanthic acids (XAs) in water. XAs endanger aquatic plants and animals, as well as human health. To the best of our knowledge, XA analysis is mainly limited to butyl xanthate. Moreover, the isomers and congeners of XAs cannot be determined separately using the existing methods. Herein, a novel method based on ultra performance liquid chromatography-tandem mass spectrometry (UPLC-MS/MS) was established to separate and analyze five XAs, namely, ethyl-, isopropyl-, *n*-butyl-, isobutyl-, and amyl-XAs, in water. Water samples were filtered through a 0.22 μm hydrophilic polytetrafluoroethylene (PTFE) membrane and directly injected into the UPLC-MS/MS instrument. Separation was performed using a Waters Acquity UPLC BEH C_18_ column (100 mm×2.1 mm, 1.7 μm) with ammonia solution (pH 11)-acetonitrile (9∶1, v/v) as the mobile phase for isocratic elution. The five XAs were detected in the negative electrospray ionization (ESI^-^) and multiple reaction monitoring (MRM) modes. An internal standard method was used for quantification. The pretreatment and UPLC-MS/MS conditions were comprehensively optimized to achieve the separation and analysis of the five XAs via direct injection. The XAs showed negligible adsorption on hydrophobic PTFE, hydrophilic PTFE, hydrophilic polypropylene, and polypropylene membranes during filtration. However, the amyl-XA showed obvious adsorption on nylon and polyether sulfone membranes. The five XAs mainly formed [M-H]^-^ parent ions in the ESI^-^ mode and the main daughter ions obtained following collisional fragmentation depended on the alkyl groups of the XAs. Increasing the pH of the ammonia solution in the mobile phase to 11 led to the isomeric separation of *n*-butyl- and isobutyl-XAs. The optimized mobile phase inhibited the tailing of the chromatographic peak of amyl-XA and effectively improved all the chromatographic peak shapes of XAs. The BEH C_18_ column was selected as the chromatographic column owing to its better compatibility with high-pH solutions compared with the T3 C_18_ column. Preservation experiments conducted over 8 d showed that the concentration of all five XAs decreased over time at room temperature; among the XAs analyzed, the concentration of ethyl-XA revealed the most significant decrease. However, the recoveries of the five XAs at 4 and -20 ℃ remained high, ranging from 101% to 105% and from 100% to 106%, respectively, on the 8th day. The preservation observed with a high concentration of XAs was similar to that found with a low concentration. The preservation time was extended to 8 days at pH 11 and 4 ℃ away from the light. No significant matrix effects were observed for the five XA samples in surface water and groundwater, but industrial sewage exerted obvious matrix inhibitory effects on ethyl- and isopropyl-XAs. Owing to the short retention times of ethyl- and isopropyl-XAs, the co-fluxed interferents in the industrial sewage depressed the MS signals. The five XAs showed good linearity in the range of 0.25-100 μg/L, with correlation coefficients greater than 0.9996. The method detection limits were as low as 0.03-0.04 μg/L, and the intra- and inter-day precisions were 1.3%-2.1% and 3.3%-4.1%, respectively. The recoveries obtained under low, medium, and high spiked levels (1.00, 20.0, 80.0 μg/L) were 96.9%-133%, 100%-107%, and 104%-112%, respectively. The corresponding RSDs were 2.1%-3.0%, 0.4%-1.9%, and 0.4%-1.6%, respectively. The optimized method was successfully applied to the analysis of XAs in surface water, groundwater, and industrial sewage. The method could separate and detect various congeners and isomers of XAs without the need for cumbersome pretreatment processes, and its advantages include smaller sample requirements, simpler operation, higher sensitivity, and longer preservation time. The proposed technique presents excellent application potential in XA environmental monitoring and water evaluation, and mineral flotation studies.

黄原酸盐(xanthate)亦称黄药,是一类结构通式为ROCSSMe(式中R为烃基,Me为Na或K)的化合物,被作为泡沫浮选捕收剂大量应用于选矿业^[1]^。黄原酸盐的烃基长度和结构决定了其对金属矿物的捕收能力和选择性,含不同烷基(如乙基、戊基、正丁基、异丁基等)的黄原酸盐常被用于不同矿物的浮选过程^[2]^。随着选矿废水的排放,黄原酸盐进入环境水体,以黄原酸(xanthic acid)分子或黄原酸根离子形式存在,生成二硫化碳(CS_2_)对水体中的水生动植物、哺乳动物等产生生长抑制、致死致畸等毒害作用^[3,4]^。乙基、异丙基、异丁基和戊基黄原酸钠对于大型蚤的半致死浓度(LC_50_)为0.35~3.7 mg/L,其中乙基黄原酸钠的毒性最强^[5]^。有报道认为黄原酸钠对哺乳动物的毒性大小与其烷基的结构和长度密切相关^[6,7]^。澳大利亚和新西兰为保护水生生物,规定水中乙基黄原酸钠的含量不得高于0.05 ng/mL^[8]^。我国《地表水环境质量标准》(GB 3838-2002)以及最新发布的《生活饮用水卫生标准》(GB 5749-2022)分别规定丁基黄原酸的标准限值为0.005 mg/L和0.001 mg/L^[9,10]^。

水中低浓度的黄原酸同系物及同分异构体的分析测试是开展水中黄原酸污染控制、保护水生态和人体健康的基础。现有的水中黄原酸分析方法主要集中在丁基黄原酸单个化合物的分析,无法实现丁基黄原酸同分异构体及多种黄原酸同系物的分析^[11⇓-13]^,主要方法包括分光光度法、气相色谱法(GC)、气相色谱-质谱法(GC-MS)、液相色谱法(LC)、液相色谱-电感耦合等离子体质谱法(LC-ICP/MS)、离子色谱-三重四极杆质谱法(IC-QQQ-MS)、液相色谱-质谱法(LC-MS)。其中,分光光度法只能测定黄原酸盐总量,且方法检出限高(0.004 mg/L)^[14]^; GC和GC-MS需要将黄原酸类物质经前处理反应生成CS_2_进行测定,无法区分黄原酸单体,同时测定结果受水样中CS_2_和氧化性物质的干扰^[15⇓-17]^。在已有的研究报道中,LC类方法局限于单个黄原酸化合物的分析^[18⇓⇓⇓-22]^。其中,LC、LC-ICP/MS和IC-QQQ-MS被用于测定水中丁基黄原酸或乙基黄原酸^[18⇓-20]^, LC-MS被用于测定水中丁基黄原酸或戊基黄原酸^[21,22]^。LC-MS具有灵敏度高、定性能力强等特点,在水中有机物分析中具有广泛的应用前景,然而目前未见其用于同时测定多种黄原酸及同分异构体的研究报道。

此外,水体样品的保存对于准确测定低浓度黄原酸至关重要。水中黄原酸的降解途径主要有两种:在酸性条件下,黄原酸降解为醇类和CS_2_等;在碱性条件下,黄原酸降解为醇类、CS_2_、 ${\mathrm{CO}}_{3}^{2-}$ 和S^2-^等^[23,24]^。普遍认为黄原酸的保存与水样pH密切相关,在酸性条件下黄原酸会迅速降解,而对于碱性条件下的降解情况,不同研究间存在一定的差异。有研究认为当水样pH>8,黄原酸降解缓慢^[25]^;另有研究认为乙基黄原酸盐在pH 9~10条件下快速降解,在pH 7~8和pH>10条件下保持稳定^[26]^。现有的保存条件主要是针对丁基黄原酸,而其他黄原酸的保存情况尚不清楚,并且对于丁基黄原酸,由于没有合适的保存条件,其保存期限普遍较短,仅为1~3 d^[11⇓-13]^。

本研究通过优化前处理和色谱、质谱分析条件及样品保存条件,建立了5种黄原酸类化合物及同分异构体的直接进样-超高效液相色谱-串联质谱法(UPLC-MS/MS),可将水样保存期限延长至8 d。该方法前处理操作简单,灵敏度高,抗干扰能力强,样品保存期限长,可有效满足水中多种黄原酸类化合物及同分异构体的准确分析。

## 1 实验部分

### 1.1 仪器、试剂与材料

超高效液相色谱(U3000,美国赛默飞科技);三重四极杆质谱仪,配备电喷雾离子源(API4000,美国AB Sciex);高纯水发生器(Direct 8,法国密理博);旋涡振荡器(QL-901,中国其林贝尔)。

乙基黄原酸钾(纯度96%,美国Aldrich), 异丙基黄原酸钾、正丁基黄原酸钾、戊基黄原酸钾(纯度分别为96%、95%、90%, 日本TCI), 异丁基黄原酸钾(纯度90%, 中国Pansine Chemical), 内标2,4-二氯苯氧乙酸-^13^C_6_(2,4-DA-^13^C_6_, 100 μg/L,德国Dr. Ehrenstorfer);乙腈(色谱纯,德国默克), 二次去离子水(密理博制水机制备)。

### 1.2 黄原酸标准溶液的配制

分别称取一定量的5种黄原酸钾溶于去离子水中,用氢氧化钠溶液调节pH至11,得到100 mg/L的黄原酸单标储备液。分别移取一定量的单标储备液,用去离子水混合稀释,用氨水溶液调节pH至11,得到10.0 mg/L的混标中间液。移取一定量的混标中间液,用去离子水稀释,用氨水溶液调节pH至11,得到1.0 mg/L的混标使用液,现用现配。

### 1.3 水样采集、保存和前处理

将水样采集至配有聚四氟乙烯垫螺旋盖的棕色玻璃瓶中,使用氢氧化钠溶液调节pH至11,于4 ℃冷藏避光保存。上机分析前用0.22 μm亲水聚四氟乙烯(PTFE)滤膜过滤水样,取1.0 mL滤液,加入2,4-DA-^13^C_6_(最终质量浓度为10.0 μg/L)混匀。

### 1.4 分析条件

#### 1.4.1 色谱条件

Waters ACQUITY UPLC BEH C_18_色谱柱(100 mm×2.1 mm, 1.7 μm), Waters ACQUITY UPLC BEH C_18_保护柱(5 mm×2.1 mm, 1.7 μm),流动相为氨水溶液(pH 11)-乙腈(9∶1, v/v),等度洗脱,流速0.3 mL/min,柱温30 ℃,进样量10 μL。

#### 1.4.2 质谱条件

电喷雾负离子(ESI^-^)扫描模式,多反应监测(MRM),喷雾电压和入口电压分别为-4500 V和-10 V,离子源加热气体温度700 ℃,离子源雾化气压力、气帘气压力、离子源辅助加热气压力和碰撞气压力分别设为413.6、275.8、413.6和55.2 kPa。母离子、子离子、去簇电压(DP)、碰撞能量(CE)、碰撞池出口电压(CXP)等质谱参数见表1。

**表1:** 5种黄原酸及同位素内标的质谱参数

Compound	Parent ion (m/z)	Daughter ion (m/z)	DP/V	CE/eV	CXP/V
Ethyl xanthic acid (EXA)	120.9	45.0^*^	-35	-20	-5
		42.9	-35	-28	-5
Isopropyl xanthic acid (i-PXA)	134.9	59.0^*^	-35	-16	-7
		57.1	-35	-28	-5
Isobutyl xanthic acid (i-BXA)	149.0	73.0^*^	-35	-14	-1
		71.1	-35	-22	-1
n-Butyl xanthic acid (n-BXA)	148.9	71.0^*^	-25	-20	-1
		72.7	-25	-12	-1
Amyl xanthic acid (AXA)	162.9	85.1^*^	-30	-22	-13
		86.9	-30	-14	-1
2,4-Dichlorophenoxyacetic acid-^13^C_6_ (2,4-DA-^13^C_6_)	225.0	166.9^*^	-50	-14	-21
		130.9	-50	-38	-1

DP: declustering potential; CE: collision energy; CXP: cell exit potential; * Quantitative ion.

## 2 结果与讨论

### 2.1 仪器分析条件的优化

采用流动注射的方式,优化了喷雾电压和入口电压、离子源温度、离子源雾化气压力、气帘气压力、离子源辅助加热气压力和碰撞气压力,以及不同黄原酸的去簇电压、碰撞能量及碰撞池出口电压等参数,最终确定了5种黄原酸的MRM参数,见表1。黄原酸类化合物在ESI^-^下生成[M-H]^-^母离子,该母离子通过碰撞碎裂主要丢失质量数为76 Da和78 Da的中性分子,形成相应的子离子。对于正丁基黄原酸和戊基黄原酸,丢失78 Da中性分子形成的子离子的丰度相对较高,而对于异丁基黄原酸、异丙基黄原酸和乙基黄原酸,丢失76 Da中性分子形成的子离子的丰度相对较高。对于不同黄原酸,分别选择丰度较高的子离子为定量离子,另一个子离子为定性离子。为了提高质谱检测的准确性,选择与黄原酸性质类似的2,4-DA-^13^C_6_作为同位素内标,采用内标法定量。

黄原酸类化合物极性强,易溶于水,通过电离和水解作用在水中以离子和分子形式存在,在液相色谱中易出现保留弱且峰形差的现象^[18,21,22]^。比较了普通的ACQUITY UPLC BEH C_18_色谱柱(100 mm×2.1 mm, 1.7 μm)和低碳载量的ACQUITY UPLC HSS T3 C_18_色谱柱(100 mm×2.1 mm, 1.8 μm)对5种黄原酸的分离效果。结果显示,2种色谱柱均可通过优化流动相改善色谱峰形,实现5种黄原酸的有效分离。黄原酸在酸性条件下遇水时易发生分解,只能在碱性流动相条件下进行色谱分离^[18,21,22]^,且BEH C_18_色谱柱的最高耐受pH(12)高于HSS T3 C_18_色谱柱(pH 8)。为保证色谱柱的使用寿命,选择BEH C_18_色谱柱用于5种黄原酸的分离。

以往研究显示,以LC或LC-MS分析丁基黄原酸或戊基黄原酸时,流动相采用pH 9.5或pH 10的氨水溶液可获得较好的色谱峰形^[18,21,22]^。分别使用超纯水(pH 6.5)、氨水溶液(pH 9.5和pH 11)为流动相,研究了流动相pH对多种黄原酸及同分异构体色谱分离的影响。结果显示,流动相pH为6.5或9.5时,正丁基黄原酸和异丁基黄原酸均无法被有效分离,但在pH 9.5条件下戊基黄原酸色谱峰的拖尾现象得到有效抑制;当pH为11时,5种黄原酸可被有效分离,且峰形较好。为提高5种黄原酸的分离效果,尤其是实现正丁基黄原酸和异丁基黄原酸的相互分离,进一步优化流动相中乙腈的比例。最终确定流动相条件为氨水溶液(pH 11)-乙腈(9∶1, v/v),等度洗脱。在此条件下对100 μg/L的5种黄原酸混合标准溶液进行分析,5种黄原酸在5 min内得到分离,相应的色谱图见图1。

**图1:**
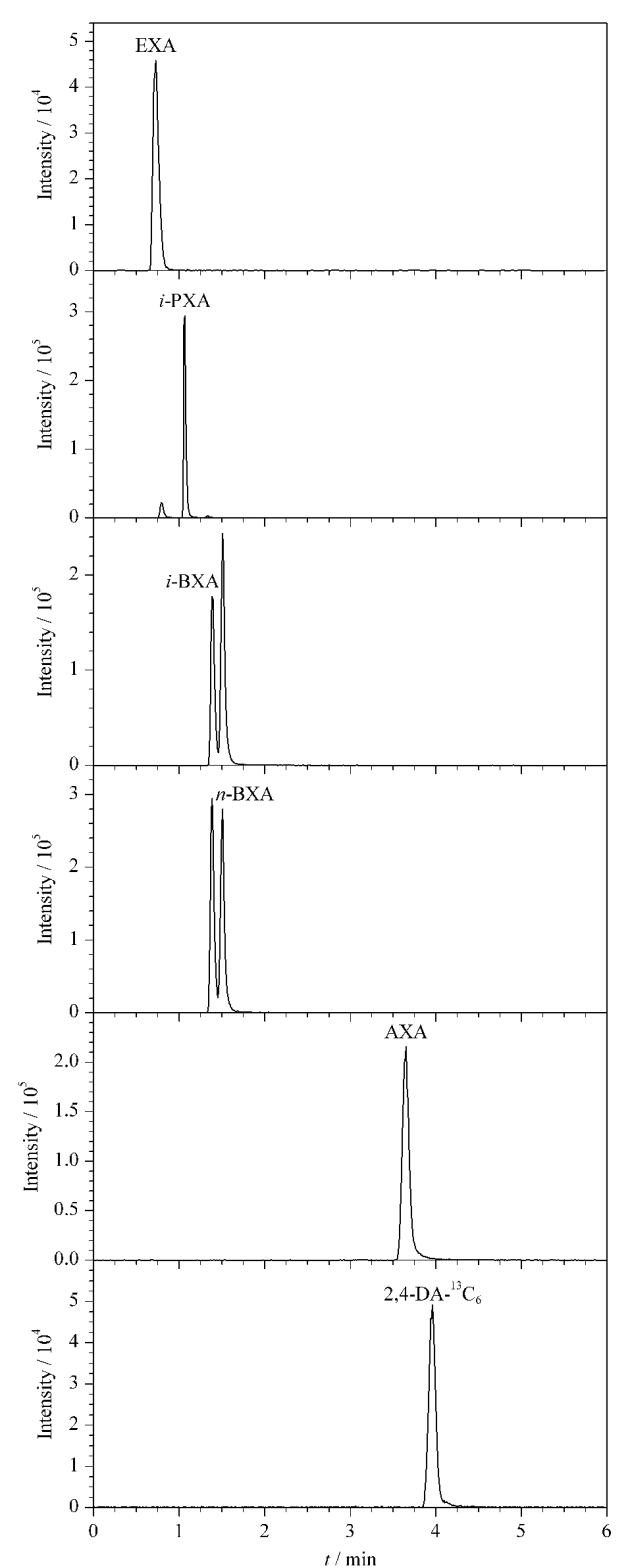
5种黄原酸混合标准溶液(100 μg/L)的色谱图

### 2.2 前处理条件的优化

采用直接进样方法,在上机前需要对水样进行过滤以去除悬浮物。配制质量浓度均为100 μg/L的5种黄原酸混合标准溶液,分别用0.22 μm孔径的6种材质滤膜(尼龙(PA)、聚醚砜(PES)、疏水性PTFE、亲水性PTFE、亲水性聚丙烯(GHP)、聚丙烯(PP))过滤,考察不同材质滤膜对水中黄原酸回收率的影响。结果显示,疏水性PTFE、亲水性PTFE、GHP、PP材质滤膜对5种黄原酸几乎没有吸附,而PA、PES材质滤膜对戊基黄原酸产生了明显的吸附作用,其回收率分别为85.3%和90.1%(相比于亲水性PTFE滤膜)。亲水性滤膜较疏水性滤膜在过滤水质样品时更易被润湿、过滤压力小,且PTFE材料具有极强的化学稳定性,因此本研究最终选用亲水性PTFE滤膜。

### 2.3 水样保存条件的确定

水样中黄原酸的保存期限与黄原酸种类、水样pH及温度有关。有研究发现3种高浓度(20 mg/L)黄原酸盐(乙基黄原酸钠、异丙基黄原酸钠、戊基黄原酸钾)在酸性条件下迅速降解产生CS_2_;在室温25 ℃、较高pH条件下,3种高浓度黄原酸盐在4.7 d内均发生不同程度的降解,其降解程度与水样pH大小密切相关,排序为pH 10<pH 8<pH 6^[24]^。此外,较低的保存温度可有效减少高浓度异丁基黄原酸钠(质量分数0.05%)降解产生CS_2_^[23]^。对于丁基黄原酸,常用方法是将水样调节至中性或弱碱性(pH 9~10),并在4 ℃下避光保存,但水样保存期限仍然较短(1~3 d)^[11⇓-13]^。同时采用较高pH(如pH 11)和较低温度(如4 ℃)的保存条件很可能延长水样保存期限。

考察了5种黄原酸(5 μg/L和100 μg/L)在不同水样(去离子水和地表水,pH 11)及不同保存温度(25、4和-20 ℃)下回收率随时间(8 d内)变化的情况,其中低水平(5 μg/L)黄原酸保存结果见图2。结果显示,在室温条件下,低加标水平的去离子水和地表水中黄原酸回收率变化情况类似。以去离子水为例,当保存温度为25 ℃时,5种黄原酸的回收率随时间的增长而下降,其中乙基黄原酸回收率下降最为显著,第8 d仅为17.8%;而其余4种黄原酸回收率随时间的增长缓慢下降,第8 d回收率为58.5%~72.1%。当保存温度变为4 ℃和-20 ℃时,5种黄原酸均保持较高的回收率,第8 d回收率分别为101%~105%和100%~106%。对于去离子水和地表水中较高水平(100 μg/L)的黄原酸,其变化情况和低水平类似。因此,确定水样保存条件为水样pH调至11,并放置在4 ℃下避光保存,在该条件下水样的保存期限可延长至8 d。

**图2:**
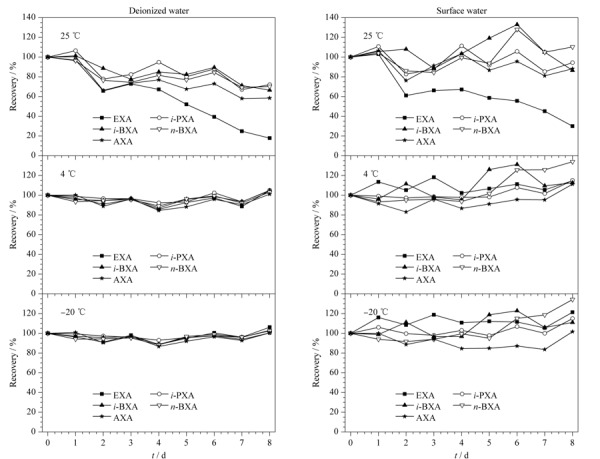
去离子水和地表水中5种黄原酸(5 μg/L)在不同温度下的回收率

### 2.4 基质效应

对于直接进样法,水体中的基质容易对目标物的质谱分析带来干扰。采用样品中黄原酸峰面积与标准溶液中黄原酸峰面积的比值来评估实际水样的基质效应(ME)。当ME等于100%时,不存在基质效应;ME小于或大于100%时,基质效应分别对应为基质抑制效应或基质增强效应。

考察了以地表水、地下水为代表的简单基质和以电子工业废水为代表的复杂基质中20.0 μg/L黄原酸的基质效应,结果见图3。地表水和地下水中5种黄原酸的ME为82.5%~117%,说明其基质效应较小;工业废水中正丁基黄原酸、异丁基黄原酸和戊基黄原酸的基质效应较小(ME为88.0%~118%),而乙基黄原酸和异丙基黄原酸有明显的基质抑制效应(ME为46.5%和68.1%),这是由于乙基黄原酸和异丙基黄原酸的色谱保留时间短,在复杂基质分析中易受到共流出杂质影响。由此可见,简单基质水体中5种黄原酸的基质效应小,而复杂基质水体中,出峰时间早的黄原酸易产生明显的基质抑制效应。通过采用内标法或延长黄原酸出峰时间,可降低复杂水体的基质效应。因此,本文采用了内标法。

**图3:**
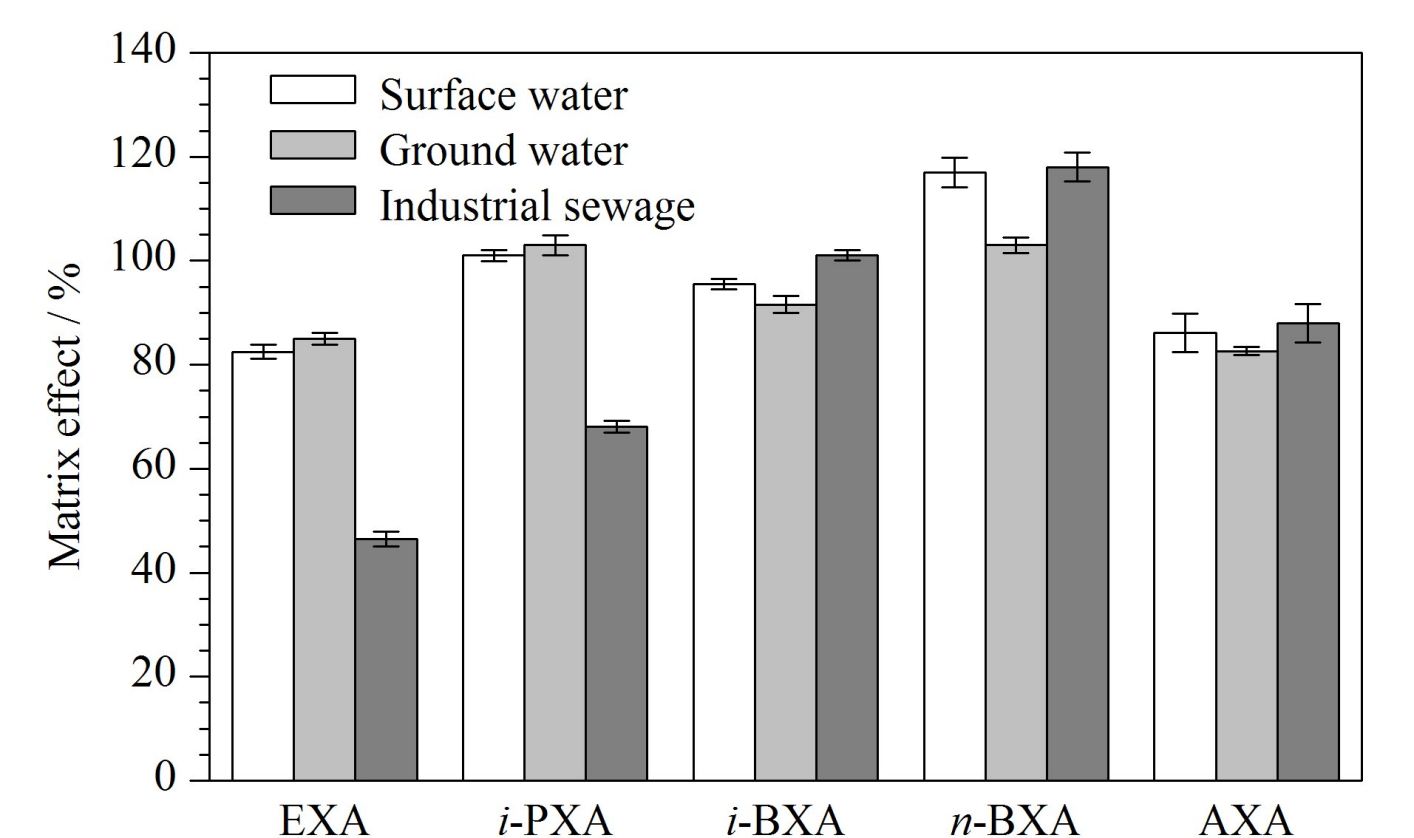
5种黄原酸在不同基质水体中的基质效应

### 2.5 方法性能

通过逐级稀释黄原酸混合标准溶液并调节pH至11,得到0.25、0.50、1.00、2.00、5.00、10.0、20.0、50.0、75.0、100.0 μg/L系列混合标准溶液,加入内标(最终质量浓度为10 μg/L)混匀,采用优化的方法分析,绘制标准曲线。参照《环境监测分析方法标准制订技术导则》(HJ 168-2020)确定方法检出限(MDL),即平行测定7次0.1 μg/L的黄原酸混合标准溶液,对结果进行统计计算。配制1.00、20.0、80.0 μg/L黄原酸混合标准溶液,分别测定其日内精密度(*n*=7)和连续7 d的日间精密度。此外,考察了去离子水低、中、高加标水平(1.00、20.0、80.0 μg/L)下5种黄原酸的回收率情况(*n*=6)。结果显示,5种黄原酸标准曲线的线性相关系数(*R*)≥0.9996, MDL为0.03~0.04 μg/L,日内精密度为1.3%~2.1%,日间精密度为3.3%~4.1%,低、中、高加标水平下的回收率分别为96.9%~133%、100%~107%和104%~112%,对应的相对标准偏差(RSD)分别为2.1%~3.0%、0.4%~1.9%和0.4%~1.6%,相关数据见表2。

**表2:** 5种黄原酸的线性相关系数、方法检出限、日内、日间精密度(*n*=7)和加标回收率(*n*=6)

Compound	R	MDL/ (μg/L)	Intra-day RSD/%	Inter-day RSD/%	1.00 μg/L			20.0 μg/L			80.0 μg/L				
					Recovery/%	RSD/%		Recovery/%	RSD/%		Recovery/%		RSD/%		
EXA	0.9998	0.04	1.6	4.1	127	2.5		100	1.0		104				1.5
i-PXA	0.9999	0.03	2.0	3.3	96.9	3.0		107	1.6		110				1.3
i-BXA	1.0000	0.04	1.4	3.5	102	2.9		104	0.4		106				1.1
n-BXA	0.9999	0.03	2.1	3.6	111	2.9		103	1.9		108				1.6
AXA	0.9996	0.03	1.3	4.1	133	2.1		107	1.4		112				0.4

### 2.6 与其他方法的比较

将该方法与标准方法及文献报道方法进行比较,结果见表3。结果显示,该方法具有取样量少(2 mL)、分析黄原酸种类多(5种)、丁基黄原酸同分异构体有效分离检测、方法检出限低(0.03~0.04 μg/L)、样品保存时间长(8 d)等显著优势,可有效满足水质分析的要求。

**表3:** 黄原酸分析方法比较

Source	Method	Sample volume/mL	Xanthic acid	Distinguish isomers or not	MDL/ (μg/L)	Storage condition
Standard	SP^[27]^	500	BXA	no	2	pH 5-6
	UVSP^[14]^	NA	BXA	no	4	pH 7, ca. 4 ℃, 3 d
	P&T-GC-MS^[28]^	5	BXA	no	0.04	pH 10, ca. 4 ℃, 1 d
	LC-MS^[29]^	1	BXA	no	0.2	pH 9-10, ≤4 ℃, 2 d
Research paper	HS-GC^[15]^	10	BXA	no	0.24	-4 ℃, 2 d
	HS-SPME-GC-MS^[16]^	10	BXA	no	0.02	pH 10, refrigeration, 1 d
	LC^[18]^	NA	BXA	no	0.8	pH 9-10
	LC-MS^[21]^	NA	BXA	no	0.08	NA
	LC-MS^[22]^	NA	AXA	no	0.114	pH 9-10, 4 ℃, ≤1 d
This study	HPLC-MS	2	EXA, i-PXA, i-BXA,	yes	0.03-0.04	pH 11, 4 ℃, 8 d
			n-BXA, AXA			

SP: spectrophotometric; UVSP: ultraviolet spectrophotometric; P&T: purge and trap; HS-GC: headspace-gas chromatography; HS-SPME: headspace solid-phase microextraction; NA: not available.

### 2.7 实际水样分析

选择地表水、地下水、电子工业废水3种实际水样进行分析,并分别进行低、中、高水平(1.00、20.0、80.0 μg/L)加标回收试验,考察方法的实际应用情况,结果见表4。5种黄原酸在地表水、地下水和电子工业废水中均未检出。5种黄原酸在地表水和地下水中的加标回收率较高,地表水中的加标回收率为80.3%~129%, RSD为0.8%~7.7%;地下水中的加标回收率为84.7%~114%, RSD为0.9%~3.6%。对于电子工业废水,正丁基黄原酸、异丁基黄原酸和戊基黄原酸的加标回收率较高,为93.6%~127%, RSD为1.3%~5.3%,而乙基黄原酸和异丙基黄原酸的加标回收率较低,仅为46.1%~79.7%, RSD为2.3%~6.3%。乙基黄原酸和异丙基黄原酸出峰时间早,容易受到共流出杂质干扰,导致其产生明显的基质抑制效应,回收率偏低。

**表4:** 5种黄原酸在3种水样中的加标回收率和相对标准偏差(*n*=6)

Compound	Background/ (μg/L)	Spiked level/ (μg/L)	Surface water			Ground water			Industrial sewage	
			Recovery/%	RSD/%		Recovery/%	RSD/%		Recovery/%	RSD/%
EXA	ND	1.00	98.2	6.6		100	3.1		79.7	3.0
		20.0	85.2	2.1		87.0	1.5		49.1	2.8
		80.0	88.5	1.7		92.6	1.4		46.1	3.2
i-PXA	ND	1.00	88.3	6.9		84.7	3.6		52.2	6.3
		20.0	111	1.6		112	2.0		74.8	4.3
		80.0	113	0.8		114	1.8		78.6	2.3
i-BXA	ND	1.00	80.3	7.7		85.4	2.3		97.9	3.5
		20.0	102	1.9		96.7	2.3		108	4.3
		80.0	105	1.4		105	1.6		117	1.3
n-BXA	ND	1.00	103	7.0		93.0	3.5		124	5.3
		20.0	123	4.1		108	2.0		124	2.6
		80.0	129	2.3		113	1.2		126	2.5
AXA	ND	1.00	101	7.2		106	1.7		127	3.3
		20.0	94.8	3.2		90.8	1.3		98.0	2.9
		80.0	103	2.0		98.1	0.9		93.6	3.0

ND: not detected.

## 3 结论

建立了5种黄原酸的直接进样-超高效液相色谱-串联质谱法,确定了适合的水样保存条件,并成功用于地表水、地下水和工业废水的分析。该方法可实现多种黄原酸及其同分异构体的分离检测,无需繁琐的前处理过程,延长了水样保存期限,具有操作简单、灵敏度高、分析速度快等优点,在水体黄原酸类化合物监测评估方面具有广阔的应用前景。
